# The Stage-Based Psychosocial Adaptation Model for Lung Transplantation (SPA-LT): A Proposed Longitudinal Conceptual Framework for Clinical Care

**DOI:** 10.3390/jcm15145378

**Published:** 2026-07-09

**Authors:** Aleksandra Stańska

**Affiliations:** Division of Quality of Life Research, Department of Psychology, Faculty of Health Sciences, Medical University of Gdańsk, Marii Skłodowskiej-Curie Street 3A, 80-210 Gdańsk, Poland; astanska@gumed.edu.pl; Tel.: +48-601-180-636

**Keywords:** lung transplantation, psychosocial adaptation, psychological functioning, quality of life, transplant psychology, psychosocial care, patient-centered care, adherence, conceptual framework, SPA-LT

## Abstract

Lung transplantation is a life-saving intervention for patients with end-stage respiratory failure, yet it involves a uniquely complex and prolonged psychosocial trajectory. Despite growing recognition of psychological factors, they are often conceptualized as secondary outcomes and assessed at isolated time points rather than across the full transplant process. This conceptual article integrates empirical findings, theoretical perspectives, and clinical expertise to examine psychological functioning and quality of life across pre-transplant, early postoperative, and long-term phases of lung transplantation, and to develop a longitudinal framework for psychosocial care. Evidence suggests that psychological functioning in lung transplantation is dynamic, stage-dependent, and closely embedded within medical processes. The pre-transplant phase is characterized by uncertainty, distress, and identity disruption; the early postoperative period by neurocognitive vulnerability, reduced agency, and acute stress responses; and long-term adjustment by chronic vigilance, fear of rejection, and challenges in role reintegration. Existing models provide valuable but incomplete perspectives, often lacking temporal specificity and insufficiently accounting for medically driven discontinuities. To address these gaps, this article introduces the Stage-Based Psychosocial Adaptation Model for Lung Transplantation (SPA-LT), a longitudinal framework conceptualizing psychological functioning as an evolving process across the transplant trajectory. Using a narrative conceptual synthesis, literature was identified through structured searches of PubMed/MEDLINE, Scopus, and Google Scholar, supplemented by citation tracking, with priority given to adult lung transplantation studies and relevant extrapolated evidence from transplantation, intensive care, and chronic illness literature. This approach integrates assessment and intervention across stages of care and emphasizes relational continuity and contextual interpretation of distress. Reframing psychological functioning as a core component of transplant outcomes may support more effective, patient-centered care and inform future research on integrated psychosocial interventions.

## 1. Introduction

Lung transplantation represents one of the most medically complex and psychologically demanding trajectories in contemporary medicine. Worldwide, lung transplantation has become an established treatment for selected patients with end-stage respiratory diseases. According to the International Society for Heart and Lung Transplantation (ISHLT) Registry, median post-transplant survival exceeds 6–7 years, although survival varies according to underlying diagnosis, recipient characteristics, and transplant era [1]. Despite continuing improvements in survival, psychological morbidity remains common throughout the transplant trajectory. Previous studies report clinically significant symptoms of anxiety and depression in approximately 20–40% of lung transplant candidates and recipients, while ICU-related distress and post-traumatic stress symptoms may occur in a substantial proportion of patients during postoperative recovery [2,3]. These findings highlight that successful transplantation involves not only graft survival but also long-term psychosocial adaptation.

Despite these distinctive features, psychological functioning in lung transplantation has often been treated as a secondary outcome, typically assessed through isolated measures of depression, anxiety, or quality of life at single time points [2,4]. Although this literature has provided valuable descriptive findings, it has rarely been integrated into a coherent understanding of adaptation across the full transplant trajectory. Consequently, psychosocial care remains frequently fragmented, reactive, and centered more on risk identification than on sustained support.

At the same time, psychological factors are closely linked to clinically meaningful outcomes, including adherence to immunosuppressive regimens, engagement in rehabilitation, patient-reported quality of life, and long-term functional recovery [3,5,6]. Yet existing frameworks, including biopsychosocial and stress and coping models, are usually applied in broad terms and may insufficiently capture the stage-specific and medically driven disruptions that characterize lung transplantation. Similarly, structured psychosocial assessments used before transplantation are often employed primarily as predictive or gatekeeping tools rather than as foundations for targeted, stage-sensitive intervention [7,8].

There is therefore a need for an integrative perspective that conceptualizes psychological functioning in lung transplantation as a stage-dependent process embedded within the medical trajectory. Such an approach shifts attention from isolated psychological endpoints to adaptation across pre-transplant distress, early postoperative instability, and long-term adjustment under chronic medical uncertainty.

The aim of this conceptual paper is twofold. First, to integrate and critically discuss existing evidence on psychological functioning and quality of life across the lung transplant trajectory. Second, to introduce the Stage-Based Psychosocial Adaptation Model for Lung Transplantation (SPA-LT), a clinically oriented framework that integrates psychosocial assessment, early postoperative experiences, intervention targets, and long-term adjustment into a coherent structure of care.

While empirical evidence directly linking psychosocial processes to long-term graft outcomes remains limited, converging findings suggest that psychological functioning may indirectly influence clinically relevant endpoints, particularly through adherence and engagement in care.

The proposed framework is derived from an integration of the published literature and informed by the author’s clinical experience as a psychologist working within a multidisciplinary lung transplant program. The clinical experience referred to in this manuscript reflects professional expertise accumulated during routine clinical practice and did not involve analysis of patient records, interviews, clinical databases, or any identifiable patient-level information. Accordingly, no new human participant data were collected or analyzed in developing the present conceptual framework.

Throughout this article, distinctions are made between findings supported directly by lung transplantation research and concepts extrapolated from broader transplantation, intensive care, chronic illness, or health psychology literature where lung transplant-specific evidence remains limited. Such extrapolation is intended to support conceptual development rather than imply equivalent levels of empirical evidence.

### Methodological Approach

This conceptual paper employed a narrative conceptual synthesis to integrate current evidence on psychosocial functioning across the lung transplantation trajectory and to develop a clinically oriented longitudinal framework. Because the primary objective was theory development and conceptual integration rather than quantitative evidence synthesis or effect estimation, a narrative approach was considered more appropriate than a systematic review.

The literature informing the proposed framework was identified through structured searches of PubMed/MEDLINE, Scopus, and Google Scholar. Searches were initially conducted between January and April 2026 and were updated prior to manuscript submission to incorporate newly published relevant studies. Google Scholar was used as a supplementary source for citation tracking and identification of additional relevant publications.

Searches combined keywords related to lung transplantation, psychological functioning, psychosocial adaptation, quality of life, psychological distress, adherence, psychosocial assessment, SIPAT, fear of rejection, post-intensive care syndrome, ICU survivors, body image, identity reconstruction, self-management, and transplant psychology.

Priority was given to peer-reviewed studies involving adult lung transplant candidates or recipients, including observational studies, qualitative studies, systematic reviews, clinical recommendations, and conceptual papers. No predefined publication date restriction was applied because the aim was conceptual synthesis rather than historical description. Earlier publications were included when they represented seminal theoretical contributions or landmark empirical studies considered essential for understanding psychosocial adaptation in chronic illness or transplantation.

When lung transplant-specific evidence was unavailable or limited, relevant literature from broader solid organ transplantation, intensive care medicine, chronic illness adaptation, and health psychology was incorporated to contextualize psychosocial processes. Such evidence is explicitly identified throughout the manuscript as extrapolated rather than lung transplant-specific.

The retrieved literature was interpreted using an integrative conceptual synthesis approach consistent with established recommendations for theory development in complex clinical phenomena [9,10]. Studies were not formally quality-appraised because the objective was conceptual integration rather than quantitative evidence synthesis. Consequently, no PRISMA procedures, formal risk-of-bias assessment, or meta-analytic methods were applied. Emerging concepts were iteratively organized according to the temporal progression of the lung transplant pathway, resulting in identification of three major psychosocial phases—pre-transplantation, early postoperative recovery, and long-term adaptation—which subsequently formed the conceptual basis of the Stage-Based Psychosocial Adaptation Model for Lung Transplantation (SPA-LT).

## 2. Lung Transplantation as a Distinct Psychosocial Context

Lung transplantation differs from other solid organ transplants not only in medical complexity but also in its distinctive psychological trajectory. Advanced respiratory failure is characterized by progressive dyspnea, functional decline, and loss of autonomy. Unlike conditions such as end-stage renal disease, lung disease often involves persistent breathlessness and awareness of limited respiratory reserve, which are closely linked to heightened anxiety, catastrophic interpretations of bodily sensations, and increased vigilance toward physical symptoms [2,11].

Chronic respiratory failure affects functioning long before transplantation becomes imminent. Oxygen dependence, reduced mobility, and repeated hospitalizations contribute to social withdrawal and identity disruption, with distress often reflecting a coherent response to sustained physiological threat rather than a comorbid condition [2,12].

The waiting period further intensifies this burden through prolonged uncertainty and limited control. Patients frequently experience a coexistence of hope and fear, including awareness that transplantation is contingent on donor death, which has been associated with anxiety, depressive symptoms, and decisional tension [2].

The perioperative and early postoperative phases introduce additional psychological challenges. Intensive care exposure, mechanical ventilation, and complex treatment regimens may lead to delirium, fragmented memory, and acute stress responses [13,14], with early recovery often experienced as disorientation, reduced agency, and bodily unfamiliarity.

Importantly, these stages are cumulative and interdependent. Pre-transplant vulnerabilities may influence acute postoperative responses, while ICU experiences may shape long-term interpretations of transplantation [13,15]. Lung transplantation should therefore be understood as a continuous psychosocial process embedded within the medical trajectory, supporting the need to conceptualize psychological functioning as a core component of transplant care and to develop stage-sensitive models of psychosocial adaptation.

## 3. Psychological Functioning Before Transplantation

The pre-transplant period constitutes a psychologically distinct stage in which distress is often closely linked to the subjective experience of dyspnea, catastrophic interpretation of breathlessness, heightened interoceptive awareness, and increased attention to bodily sensations [2,16]. Patients face sustained medical uncertainty, progressive functional decline, and prolonged “conditional survival,” in which deterioration and potential rescue coexist [2]. Psychological functioning at this stage should therefore not be understood as a static baseline but as a dynamic process shaped by fluctuating symptoms and repeated clinical transitions [4]. This phase represents a critical window for identifying vulnerabilities and initiating psychosocial support that can extend into the post-transplant trajectory [2].

### 3.1. Emotional Distress and Psychological Burden During Listing

Emotional distress during listing commonly includes anxiety, depressive symptoms, and sleep disturbance, largely driven by uncertainty and reduced perceived control [3,16]. In this population, psychological distress is often closely linked to the subjective experience of respiratory symptoms, particularly dyspnea-related threat perception, catastrophic interpretation of breathlessness, and heightened attention to bodily sensations [11,12]. Anxiety symptoms, illness-related worry, perceived dyspnea severity, sleep disturbance, and depressive symptoms frequently fluctuate with changes in respiratory status, exacerbations, hospitalization, and waiting-list uncertainty, forming an episodic pattern that may be insufficiently captured by cross-sectional assessment.

Beyond affective symptoms, transplant listing is characterized by decisional ambivalence and existential tension. Patients may simultaneously experience hope, fear, and guilt related to transplantation, reflecting the paradox of survival through organ donation [2,17]. This ambivalence is clinically relevant, as it may influence engagement with treatment and participation in decision-making [18].

Identity disruption represents another core dimension of this stage. Progressive respiratory failure often entails loss of autonomy and valued roles, accompanied by anticipatory loss of one’s prior functioning and future expectations [2,19]. Such reactions are best understood as contextually coherent responses to sustained threat rather than as indicators of poor motivation or maladjustment.

### 3.2. Health Behaviors and Psychosocial Risk

Pre-transplant psychosocial risk extends beyond psychiatric symptoms to include health behaviors, environmental stability, and relational context [18]. Behaviors such as nicotine use, alcohol consumption, and adherence-related patterns are clinically relevant not only because of their medical implications but also as indicators of coping processes and broader social determinants.

Importantly, adherence-related vulnerability should not be conceptualized solely as an individual trait. It may reflect cognitive burden, health literacy, emotional distress, or unstable life circumstances [18]. Similarly, social support and environmental stability function as key contextual factors shaping both psychological functioning and transplant readiness [20].

From a clinical perspective, psychosocial risk is best understood as multidimensional, encompassing affective functioning, coping style, health behaviors, and social resources [7]. Crucially, many of these domains represent modifiable targets rather than fixed predictors.

### 3.3. Psychosocial Assessment Tools: Strengths, Limitations, and the “Assessment–Intervention Gap”

Structured psychosocial assessments, such as the Stanford Integrated Psychosocial Assessment for Transplantation (SIPAT), are widely used to support clinical decision-making and standardize evaluation domains [7,21]. They enhance comprehensiveness and facilitate communication within multidisciplinary teams [22,23].

However, several limitations are particularly relevant in lung transplantation. Psychosocial risk scores are often interpreted as static, despite the dynamic and context-dependent nature of many domains [8]. Additionally, categorical classifications (e.g., “low-risk” vs. “high-risk”) may obscure the distinction between modifiable vulnerabilities and structural constraints [18].

Most importantly, assessment does not automatically translate into intervention. In many settings, psychosocial evaluation functions primarily as a gatekeeping mechanism, with limited resources for longitudinal support. This creates an “assessment–intervention gap,” in which identified vulnerabilities remain insufficiently addressed [2].

Bridging this gap requires shifting from risk profiling toward stage-sensitive psychosocial care pathways that integrate assessment with targeted intervention and continuity of care [6].

As illustrated in Table 1, psychosocial domains identified during pre-transplant evaluation should not be conceptualized merely as predictors of eligibility but as clinically meaningful intervention targets. A shift from categorical risk labeling toward individualized, stage-sensitive support strategies may reduce the gap between assessment and care. Such an approach reframes psychosocial vulnerability as modifiable rather than deterministic, aligning psychological evaluation with the broader goals of transplant medicine. Table 1 represents an original conceptual synthesis developed by the author based on the literature reviewed in the present article and is intended to organize clinically relevant psychosocial domains rather than reproduce an existing framework.

## 4. Early Post-Transplant Period: Acute Psychological Challenges

The early postoperative period following lung transplantation represents one of the most psychologically unstable and medically intensive phases of the transplant trajectory [24,25,26]. Rather than a straightforward transition to recovery, this stage is frequently characterized by neurocognitive vulnerability, emotional dysregulation, and disruption of bodily and autobiographical continuity. The intensive care environment functions not only as a site of physiological stabilization but also as a context in which acute psychological responses emerge at the intersection of surgical trauma, pharmacological exposure, and existential stress.

### 4.1. Delirium, Confusion, and Acute Stress Responses

Delirium is a common and clinically significant phenomenon in the early post-transplant period, reflecting the combined effects of critical illness, mechanical ventilation, metabolic disturbances, and pharmacological exposure [27,28,29,30]. Clinically, it manifests as fluctuating attention, disorientation, perceptual disturbances, and emotional lability.

Pharmacological management, including opioid-based analgesia, further contributes to this neurocognitive vulnerability, requiring a balance between adequate symptom control and preservation of cognitive stability [31,32].

Acute stress responses—such as fear, agitation, dissociation, or heightened emotional reactivity—are also common. Importantly, these reactions should be understood as contextually coherent responses to sensory overload, impaired communication, and loss of orientation, rather than automatically interpreted as psychiatric pathology [13].

### 4.2. ICU-Related Psychological Sequelae

The ICU environment introduces multiple stressors, including continuous monitoring, limited mobility, sleep disruption, and fluctuating levels of consciousness, all of which may disrupt the patient’s sense of temporal and experiential continuity [33]. Fragmented or distorted memories of this period are common and may contribute to later distress [13].

Evidence from critical care populations indicates that ICU exposure is associated with an increased risk of longer-term psychological sequelae, including symptoms consistent with post-traumatic stress, depression, and anxiety [14,34]. In lung transplant recipients, this vulnerability may be amplified by the cumulative burden of pre-transplant illness. Importantly, the subjective interpretation of the ICU experience may shape subsequent adaptation, functioning as a psychological inflection point within the broader trajectory [35].

### 4.3. Loss of Agency and Bodily Alienation

The early postoperative phase is also marked by profound alterations in perceived agency and embodiment. Patients transition abruptly from anticipatory waiting to a state of physical dependency, often characterized by limited communication, restricted mobility, and reliance on medical staff [26]. Even as respiratory function improves, the body may feel unfamiliar and medically dominated.

This can result in bodily alienation, where the transplanted organ is experienced as both life-saving and foreign [36,37,38]. Patients may struggle to integrate their pre- and post-transplant identities, with reactions such as detachment or ambivalence reflecting ongoing identity reconstruction rather than pathological processes.

### 4.4. Early PTSD-like Symptoms and Emotional Dysregulation

In the weeks following transplantation, some patients exhibit symptoms resembling early post-traumatic stress responses, including intrusive memories, hyperarousal, and avoidance [3,39]. Emotional dysregulation may also be present.

A key clinical challenge is differentiating transient, context-dependent stress reactions from persistent trauma-related psychopathology. Early instability often reflects the psychological integration of extreme medical experiences rather than the onset of a primary psychiatric disorder [2]. Accordingly, assessment should emphasize symptom persistence and functional impact over time.

Importantly, early postoperative psychological reactions emerge during a period of ongoing neurobiological recovery characterized by residual effects of sedation, fluctuating cognition, disrupted sleep, pain, and physiological instability. Consequently, intrusive recollections, vivid dreams, emotional lability, transient avoidance of distressing ICU memories, or heightened arousal may represent normative responses to critical illness rather than evidence of established PTSD. Premature psychiatric labeling may therefore overpathologize adaptive recovery processes and overlook the dynamic nature of psychological adjustment during the immediate postoperative period.

Not all intrusive memories following transplantation indicate the development of PTSD. Many recipients initially experience fragmented recollections, vivid dreams, emotional lability, or transient hyperarousal that gradually diminish during recovery. Clinical concern should increase when trauma-related symptoms persist beyond one month after transplantation, consistent with DSM-5 temporal criteria for PTSD, particularly when accompanied by persistent avoidance, negative alterations in cognition and mood, hyperarousal, and clinically significant impairment in rehabilitation, interpersonal functioning, or engagement with follow-up care. Early differentiation therefore requires repeated longitudinal psychological assessment rather than reliance on a single cross-sectional evaluation.

Repeated assessment is particularly important because symptom trajectories may diverge over time. While many recipients demonstrate gradual emotional integration as cognitive functioning improves and medical stability increases, a subgroup develops persistent trauma-related symptoms associated with impaired rehabilitation, reduced quality of life, avoidance of medical follow-up, or difficulties in social reintegration. Within the SPA-LT framework, emphasis is therefore placed not only on symptom severity but also on symptom persistence, functional consequences, and developmental context across recovery.

A distinctive feature of the proposed SPA-LT framework is that these early trauma-related reactions are conceptualized as stage-specific adaptation processes embedded within the broader transplant trajectory rather than isolated psychiatric complications. This longitudinal perspective emphasizes repeated psychosocial assessment across recovery and supports interpretation of distress within its evolving medical and psychosocial context.

### 4.5. Reframing Early Postoperative Reactions

Early postoperative psychological phenomena should be conceptualized as part of the adaptation process rather than as isolated complications. Situating delirium, acute stress, and emotional instability within a longitudinal framework enables more proactive and integrated care. This perspective supports early psychological presence in the ICU, coordination with medical management, and structured follow-up aimed at facilitating integration of the transplant experience.

The key psychosocial domains characterizing the early postoperative period, along with their clinical meaning and potential intervention targets, are summarized in Table 2.

Table 2 represents an original conceptual synthesis developed by the author based on the narrative integration of lung transplantation literature, evidence from intensive care medicine, post-intensive care syndrome, and transplantation psychology. The table was developed by organizing stage-specific psychosocial domains identified during the conceptual synthesis into clinically meaningful categories and is intended as a conceptual framework rather than a validated classification system.

## 5. Long-Term Psychological Adjustment After Lung Transplantation

Long-term survival after lung transplantation does not automatically translate into psychological stability or restored quality of life. Despite improved respiratory function, patients remain within a context of chronic medical surveillance, immunosuppression, and ongoing awareness of graft vulnerability [40]. Long-term adjustment therefore reflects the interplay between physical recovery, emotional integration, and identity reconstruction under persistent uncertainty [40].

Adaptation should not be conceptualized as a linear progression from illness to recovery, but as a dynamic process marked by alternating periods of stability and vulnerability, including complications and fear of rejection [41,42]. This non-linear trajectory is essential for interpreting long-term outcomes [42,43].

### 5.1. Quality of Life Outcomes

Quality of life (QoL) is a key outcome in lung transplantation and often interpreted as an indicator of success [44]. While physical domains such as dyspnea and functional capacity typically improve, gains in emotional and social functioning are less consistent [43,45].

Vulnerable domains include emotional functioning, fatigue, cognition, and role participation [42,46]. Medication side effects, body image changes, sexual dysfunction, and ongoing medical demands may limit perceived benefits [46,47]. QoL measures also often fail to capture the ambivalence of post-transplant experience, combining gratitude with chronic anxiety about graft failure [40,48].

Interpretation is further limited by reliance on generic instruments, cross-sectional designs, and survivorship bias, which may obscure temporal variability and transplant-specific concerns [42,43,48]. QoL outcomes therefore require contextualization within the broader psychosocial trajectory [40,41].

### 5.2. Emotional Disorders and Ongoing Psychological Burden

Depressive and anxiety symptoms remain prevalent in a subset of recipients [3], often reflecting chronic vigilance and uncertainty rather than pre-transplant symptom patterns [40,48].

Fear of rejection is a central and persistent concern. Even minor symptoms or routine monitoring may trigger anticipatory anxiety, contributing to emotional exhaustion and reduced flexibility [48]. Some patients describe a state of “conditional survival,” with future planning constrained by perceived graft vulnerability [40,41].

Distinguishing adaptive vigilance from maladaptive anxiety is clinically essential. While monitoring supports adherence, escalation into rumination or avoidance may require intervention [3,48]. Importantly, distress–outcome relationships are complex and not strictly linear [3].

Long-term support does not necessarily require formal psychotherapy. Accessibility of psychological support, relational continuity, and validation of emotional experiences may significantly reduce distress [40].

### 5.3. Functioning in Everyday Life

Long-term adjustment is reflected in everyday functioning, including work, relationships, autonomy, and social participation. Despite improved physical capacity, reintegration into prior roles is often incomplete [47,49].

Return to work may be limited by fatigue, cognitive effects, or altered self-perception [46,49]. Interpersonal dynamics may shift, particularly when prior caregiving roles persist [50,51,52]. Sexual functioning and body image also remain under-addressed but clinically relevant domains [47].

For many recipients of working age, successful return to employment represents an important milestone of psychosocial recovery rather than merely an economic outcome. Vocational rehabilitation, gradual workplace reintegration, flexible employment arrangements, and collaboration between transplant teams, employers, and occupational health professionals may facilitate sustainable return to work. Despite its clinical relevance, vocational rehabilitation remains underrepresented in the lung transplantation literature and should receive greater attention within integrated psychosocial care pathways. Because many lung transplant recipients are of working age, successful vocational reintegration may constitute one of the most meaningful indicators of long-term psychosocial recovery, reflecting restoration of autonomy, social identity, and participation rather than employment alone.

Adherence should be conceptualized as a relational construct shaped by trust and partnership with the medical team. Patients are more likely to engage in treatment when they experience themselves as active participants in care [40]. Broader evidence suggests that self-management is influenced by social support, health literacy, resilience, and illness uncertainty [53,54].

Although SPA-LT primarily focuses on the psychosocial adaptation of transplant recipients, caregiver functioning represents an important contextual factor throughout the transplant trajectory. Family members frequently contribute to treatment organization, medication management, rehabilitation, emotional regulation, and long-term self-management, while simultaneously experiencing their own psychological burden. Future development of SPA-LT should therefore consider dyadic and family-level adaptation processes.

Many long-term difficulties do not require intensive psychotherapy but rather relational continuity, validation, and accessible support embedded within the transplant setting [40,55].

Long-term psychosocial adaptation domains and their clinical implications are summarized in Table 3.

## 6. Existing Conceptual Models and Their Limitations

Psychosocial research in transplantation has been shaped by broad health psychology frameworks, including the biopsychosocial model, stress and coping theories, and quality-of-life (QoL) paradigms. Drawing on selected empirical studies and theoretical models, the following section critically examines existing conceptual approaches and identifies their relevance and limitations for lung transplantation. These approaches support inclusion of psychological and social variables in clinical care and highlight their relevance for outcomes such as adherence and patient-reported well-being [18].

However, lung transplantation follows a discontinuous trajectory marked by progressive respiratory failure, prolonged uncertainty, ICU exposure, and long-term survivorship under chronic medical monitoring. Psychosocial experiences therefore evolve across distinct stages rather than a linear post-surgical pathway [2,40], limiting the applicability of general models.

### 6.1. The Biopsychosocial Model: Integration Without Temporal Specificity

The biopsychosocial model provides a rationale for assessing psychological symptoms, health behaviors, and social context [18], and aligns with evidence linking psychosocial factors to outcomes [56].

However, it is typically applied as a static framework. In lung transplantation, the clinical meaning of psychosocial variables varies across stages. Early postoperative functioning is strongly influenced by ICU-related physiological and neurocognitive factors [57], while uncertainty and identity disruption evolve over time [2]. This limits its usefulness for stage-specific intervention planning.

### 6.2. Stress and Coping Frameworks: Adaptation Beyond Individual Agency

Stress and coping models emphasize appraisal, perceived control, and coping strategies. Evidence supports distinct trajectories of distress linked to symptom burden and perceived control, as well as roles of resilience and social support [58,59].

In lung transplantation, however, the stressor is prolonged, unstable, and often beyond individual control. Adaptation cannot be understood solely as an individual process, particularly during early postoperative phases marked by sedation, delirium risk, and reduced agency [57]. In such contexts, adaptation is shaped by medically structured environments.

Some vigilance may be adaptive, supporting adherence and self-management [3,48]. Distinguishing adaptive vigilance from maladaptive anxiety remains clinically essential.

### 6.3. Quality of Life Models: Measurement Without Mechanism

QoL frameworks are central to evaluating transplant outcomes. Physical functioning often improves, while some domains remain impaired or decline over time [41,42,43].

However, QoL measures are primarily descriptive and do not explain mechanisms of adaptation. Physical improvement does not necessarily reflect emotional integration, and stable scores may coexist with ongoing uncertainty or episodic distress [40]. Qualitative research further indicates ongoing changes in identity and functioning that are not captured by cross-sectional measures.

QoL should therefore be interpreted as an outcome embedded within broader psychosocial processes rather than a proxy for adaptation.

#### Critical Synthesis: Toward a Longitudinal, Medically Embedded Model

Existing frameworks provide complementary but incomplete perspectives. The biopsychosocial model lacks temporal specificity, stress and coping theories emphasize individual processes while underestimating reduced agency, and QoL models capture outcomes without mechanisms.

An integrative framework is needed that accounts for longitudinal progression, medically driven discontinuities, and the embeddedness of psychological processes within treatment contexts. In lung transplantation, adaptation is best understood as a staged, dynamic process shaped by uncertainty and medical instability [2,48].

Although numerous studies have described psychosocial problems before and after lung transplantation, existing literature has primarily focused on individual domains such as anxiety, depression, adherence, quality of life, fear of rejection, caregiver burden, or self-management. Likewise, current theoretical approaches—including the biopsychosocial model, stress and coping frameworks, and quality-of-life models—provide valuable perspectives but are not specifically organized around the temporal progression of the lung transplant trajectory.

Importantly, SPA-LT does not propose new psychosocial constructs. Rather, its novelty lies in organizing previously described psychosocial domains into a longitudinal, transplant-specific clinical framework that explicitly links stage-specific psychological tasks, psychosocial assessment, postoperative neurocognitive vulnerability, identity reconstruction, continuity of care, and intervention planning across the entire lung transplant trajectory (Table 4).

## 7. Gaps in Current Psychosocial Care

Despite growing recognition of the importance of psychological functioning in transplantation, psychosocial care in lung transplant settings remains inconsistently structured and often reactive rather than longitudinally integrated. This gap between conceptual acknowledgment and clinical implementation represents a key unmet need in transplant care.

### 7.1. Fragmented Psychological Support Across Transplant Stages

In many centers, psychological involvement is concentrated at the pre-transplant evaluation stage, where assessments are used to determine candidacy and identify psychosocial risk factors. Following transplantation, support often becomes episodic or crisis-driven rather than systematically embedded across stages of care.

This approach overlooks the stage-specific nature of psychological demands. Pre-transplant uncertainty, acute postoperative neurocognitive instability, and long-term adaptation to chronic medical surveillance represent distinct phases requiring different forms of support. Without structured continuity, patients may experience reduced support during periods of heightened vulnerability, including ICU recovery or complications.

The absence of stage-sensitive care pathways also limits early intervention, particularly in patients who do not meet formal psychiatric criteria but experience clinically meaningful distress [2,40].

### 7.2. Dominance of Screening over Intervention

Psychosocial screening is widely implemented in transplant settings, reflecting legitimate concerns regarding adherence, substance use, and social stability. However, identification of risk does not consistently lead to structured intervention.

As a result, psychosocial assessment may function primarily as a gatekeeping process rather than as the foundation of individualized care planning [7,8]. This imbalance risks reinforcing a deficit-oriented perspective, in which patients are categorized by risk level without corresponding investment in modifiable protective factors.

### 7.3. Limited Integration of Psychologists into Transplant Teams

Although multidisciplinary collaboration is widely endorsed, the degree of psychological integration varies substantially across centers. In some settings, psychologists are involved longitudinally; in others, their role is limited to isolated consultations.

Limited integration restricts continuity of care and reduces the ability to contextualize emotional and behavioral responses within the broader adaptation process. It may also reinforce hierarchical models of care in which patients are positioned as passive recipients rather than active partners.

Importantly, the therapeutic relationship itself functions as a mechanism of change. Evidence consistently shows that relational quality influences engagement, adherence, and clinical outcomes [3,60]. Without continuity, this mechanism remains underutilized.

### 7.4. Consequences for Patients and Long-Term Outcomes

The combined effect of fragmented support, screening-focused practice, and limited integration extends beyond immediate distress. Patients may hesitate to disclose difficulties, disengage from communication, or internalize distress as personal failure.

Over time, these processes may contribute to reduced trust, avoidance of care, and adherence challenges. Such outcomes are not solely attributable to individual vulnerability but are shaped by structural and relational gaps in care [2,3].

Addressing these gaps does not require universal long-term psychotherapy. Rather, integrated and stepped psychosocial care models—combining routine screening with accessible support and targeted intervention—appear sufficient to improve both quality of life and clinical outcomes [61].

Embedding psychosocial care within a longitudinal, relational framework shifts the focus from reactive management to proactive partnership. In this model, communication quality and relational trust become integral components of treatment rather than secondary considerations [60,62].

## 8. Toward the Stage-Based Psychosocial Adaptation Model for Lung Transplantation (SPA-LT)

The preceding sections have highlighted the dynamic, medically embedded, and discontinuous nature of psychological functioning in lung transplantation, as well as the structural limitations of existing conceptual models and clinical practices. In response to these gaps, the present article proposes the Stage-Based Psychosocial Adaptation Model for Lung Transplantation (SPA-LT), a longitudinal psychosocial adaptation framework that situates psychological processes within the temporal architecture of the transplant trajectory and integrates assessment, intervention, and relational continuity into a coherent model of care. Rather than conceptualizing psychological outcomes as isolated variables, the proposed framework views adaptation as an evolving, stage-dependent process shaped by cumulative medical experiences, relational context, and evolving identity reconstruction [2,63,64].

### 8.1. Core Assumptions of the Proposed Framework

#### 8.1.1. Psychological Adaptation as a Dynamic, Stage-Dependent Process

At the core of the framework lies the assumption that psychological adaptation in lung transplantation is neither static nor uniformly progressive. Instead, it unfolds across distinct medical stages—pre-transplant listing, acute postoperative recovery, and long-term survivorship—each characterized by specific stressors, vulnerabilities, and adaptive tasks [64,65].

Psychological distress at any given stage should be interpreted within its clinical context. Anxiety during listing may reflect existential uncertainty and illness-related unpredictability [58]; confusion in the ICU may be driven by neurocognitive instability and delirium-related processes [24,29]; hypervigilance during follow-up may emerge from chronic fear of rejection [48]. These responses are not interchangeable and cannot be meaningfully reduced to trait-level vulnerability.

Recognizing stage dependency allows clinicians to differentiate between contextually coherent stress reactions and persistent maladaptive patterns, thereby preventing both under-recognition and over-pathologization [63].

Although the SPA-LT stages are presented separately for conceptual clarity, they are hypothesized to be developmentally interconnected. Pre-transplant psychosocial vulnerabilities—including emotional distress, illness burden, limited social support, health literacy, or maladaptive coping—may influence psychological responses during the early postoperative period by affecting cognitive appraisal, perceived control, and engagement in recovery. Likewise, early postoperative experiences, particularly delirium, fragmented ICU memories, acute stress reactions, and loss of agency, may shape subsequent identity reconstruction, fear of rejection, self-management, and long-term quality of life. Adaptation is therefore conceptualized as a cumulative process in which experiences from earlier stages influence subsequent adjustment while remaining modifiable through stage-sensitive psychosocial intervention.

Within SPA-LT, psychosocial adaptation is conceptualized as a process linking antecedent vulnerabilities (e.g., pre-transplant psychosocial risk, illness burden, social resources), stage-specific adaptation processes (e.g., uncertainty, ICU-related experiences, identity reconstruction, relational continuity), and clinically relevant outcomes, including emotional adjustment, quality of life, self-management, and adherence. The trajectory may be modified by contextual factors such as medical complications, caregiver support, health literacy, and socioeconomic circumstances.

The proposed stages represent a general conceptual trajectory rather than a universal sequence. Individual adaptation may vary according to underlying diagnosis, urgency of transplantation, previous psychiatric history, age, socioeconomic circumstances, health literacy, cultural context, and availability of social support.

#### 8.1.2. Continuity of Care from Pre-Transplant to Long-Term Follow-Up

A second core assumption is the necessity of longitudinal continuity in psychosocial care. Psychological processes in the early postoperative period are often influenced by pre-transplant vulnerabilities, just as long-term adjustment may be shaped by ICU experiences and post-intensive care syndrome [15,66].

Within the proposed framework, psychosocial support is conceptualized as a continuous thread extending from initial evaluation through long-term follow-up. Continuity does not imply constant high-intensity intervention; rather, it entails sustained availability, relational familiarity, and the capacity to contextualize new difficulties within the patient’s broader trajectory [4,40].

### 8.2. Key Components

#### 8.2.1. Psychosocial Risk as a Starting Point, Not a Label

Structured psychosocial assessment remains essential in transplantation [7,8,21]. However, within this framework, risk profiling serves as an entry point for individualized planning rather than as a categorical determinant of suitability. Psychosocial risk factors—such as limited support, substance use patterns, or affective instability—are conceptualized as modifiable vulnerabilities embedded within a broader life context [7,8].

This reframing shifts the focus from gatekeeping to proactive support. It acknowledges that many pre-transplant risk domains can be addressed through targeted intervention, relational reinforcement, and environmental stabilization. Risk thus becomes dynamic and revisable, rather than fixed [67].

#### 8.2.2. Stage-Specific Psychological Needs

Each transplant stage is associated with distinct psychological tasks:

- During listing, the primary needs include emotional containment and coping with uncertainty [2,68].
- In the early postoperative period, stabilization and integration of ICU experiences become central [13,24].
- In long-term follow-up, identity reconstruction, chronic vigilance, and renegotiation of roles gain prominence [47,63].

Stage-specific awareness enables the development of structured care pathways that anticipate predictable vulnerabilities rather than responding solely to acute crises.

#### 8.2.3. Role of the Psychologist as an Integrated Team Member

A defining feature of the proposed framework is the integration of the psychologist as a consistent member of the transplant team. The psychologist’s function extends beyond evaluation and crisis management to include longitudinal monitoring, communication facilitation, and meaning-oriented support [62].

Importantly, integration enhances not only patient outcomes but also team functioning. Psychologists can contextualize adherence challenges, emotional reactions, or interpersonal tensions within a developmental adaptation process rather than interpreting them solely as compliance failures. Such integration fosters a relational climate in which patients are approached as partners in care rather than passive recipients of medical directives [60].

Table 5 is intended as a pragmatic operational guide rather than a prescriptive clinical protocol. The proposed assessment points, tools, and intervention targets should be adapted to local transplant pathways, available psychosocial resources, and patient-specific clinical needs.

**Figure 1:**
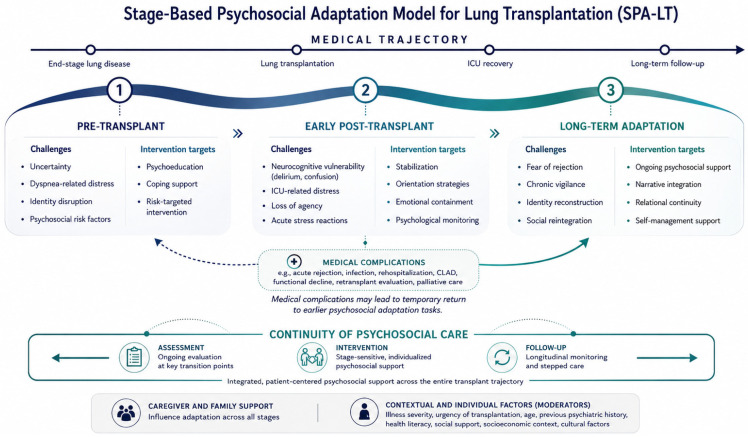
Stage-Based Psychosocial Adaptation Model for Lung Transplantation (SPA-LT). The model illustrates psychological adaptation as a dynamic, stage-dependent process across the transplant trajectory, including pre-transplant, early postoperative, and long-term phases. Each stage is associated with specific psychosocial challenges and corresponding intervention targets, embedded within the broader medical trajectory and supported by continuity of psychosocial care. Intervention targets represent conceptually derived, stage-sensitive clinical recommendations informed by available evidence rather than universally validated intervention protocols.

The proposed stage-based psychosocial adaptation model for lung transplantation is illustrated in Figure 1.

The framework is intentionally recursive rather than strictly linear. Medical complications, graft dysfunction, hospitalization, or retransplant evaluation may return patients to earlier patterns of psychological adaptation, highlighting the dynamic nature of the transplant trajectory.

The model represents a conceptual framework derived from integration of existing literature and clinical experience rather than empirical model testing.

### 8.3. Clinical Relevance

#### 8.3.1. Implications for Transplant Teams

Operationalizing a longitudinal psychosocial adaptation framework encourages transplant teams to transition from episodic consultation toward structured, stage-sensitive psychosocial care pathways. This may include routine psychological check-ins during key transitions, systematic debriefing after ICU discharge, and explicit normalization of emotional complexity in long-term follow-up [61].

Importantly, implementation does not necessitate universal psychotherapy for all recipients. In many cases, the most impactful intervention is the explicit presence of psychological expertise within the team and the clear communication that emotional concerns are legitimate components of transplant care [4].

The awareness that support is accessible—and that distress can be discussed without jeopardizing medical standing—may significantly reduce avoidance and concealment behaviors.

#### 8.3.2. Potential Impact on Patient-Reported Outcomes and Adherence

By reframing psychological functioning as integral rather than peripheral, the proposed framework has potential implications for patient-reported outcomes, adherence, and relational trust. Patients who experience continuity, partnership, and transparency are more likely to engage consistently with complex medical regimens [3,5,69].

Adherence, within this perspective, is understood not merely as behavioral compliance but as a relationally embedded construct influenced by trust, perceived respect, and collaborative decision-making. Strengthening relational quality within transplant teams may therefore function as an indirect yet powerful determinant of long-term outcomes [20].

#### 8.3.3. Potential Hypotheses Derived from SPA-LT

The proposed framework is intended to generate empirically testable hypotheses rather than provide definitive causal explanations. Examples include:

- Greater pre-transplant psychosocial vulnerability may predict higher levels of psychological distress during early postoperative recovery.
- ICU-related psychological experiences may partially mediate the association between pre-transplant vulnerability and long-term psychosocial adjustment.
- Continuity of psychosocial care may moderate the relationship between early postoperative distress and long-term quality of life.
- Successful identity reconstruction may facilitate adherence, social reintegration, and patient-reported well-being independently of physical recovery.

## 9. Implications for Clinical Practice and Future Research

### 9.1. Recommendations for Integrated Psychosocial Care

The proposed longitudinal psychosocial adaptation framework has direct implications for clinical practice in lung transplantation. First, psychosocial care should be conceptualized as a structured, stage-sensitive pathway rather than an episodic or crisis-driven intervention. This involves embedding psychological expertise across the transplant trajectory—from candidacy evaluation to long-term follow-up—while adapting the intensity and focus of support to stage-specific demands.

Second, psychosocial assessment should function as the foundation for individualized care planning rather than solely as a screening mechanism. Identified vulnerabilities, such as emotional distress, health behavior risk, or limited social stability, should trigger structured follow-up and targeted support rather than remain static descriptors within medical documentation. Current evidence-based recommendations emphasize the necessity of multidisciplinary involvement of mental health professionals throughout the transplant process and demonstrate that psychosocial interventions can improve adherence and clinical outcomes [70].

Patients presenting with persistent depressive symptoms, suicidal ideation, severe anxiety, psychotic symptoms, clinically significant substance use, severe nonadherence, or persistent trauma-related symptoms should be referred for specialist psychiatric and/or psychological assessment alongside ongoing transplant care.

Third, transplant centers should explicitly communicate to patients that emotional reactions are expected and legitimate components of the transplant journey. The normalization of psychological complexity, combined with visible availability of support within the center, may reduce avoidance, concealment of distress, and relational disengagement. The importance of therapeutic alliance and trust in healthcare relationships has been consistently linked to improved patient engagement and outcomes [60,71,72].

From a team perspective, closer integration of psychologists may enhance interdisciplinary communication, contextualize adherence challenges, and support relational trust between patients and providers. Interprofessional teamwork has been identified as a core mechanism enabling effective psychosocial care in transplantation settings [62]. In this sense, psychosocial integration is not an adjunct to medical care but a structural component of comprehensive transplant management.

#### Practical Implementation of SPA-LT

Implementation of SPA-LT does not necessarily require continuous psychological therapy throughout the transplant trajectory. Instead, the framework proposes stage-sensitive psychosocial monitoring embedded within routine multidisciplinary care. Potential implementation strategies include structured psychosocial assessment during transplant evaluation, psychological review during ICU recovery and hospital rehabilitation, routine psychosocial follow-up during outpatient care, and stepped referral to specialist psychological or psychiatric services when clinically indicated. Depending on local resources, these functions may be delivered collaboratively by transplant psychologists, psychiatrists, specialist nurses, social workers, rehabilitation professionals, or external psychological services, including telehealth where appropriate.

### 9.2. Priorities for Future Empirical Studies

Despite growing interest in psychological outcomes, significant gaps remain in the empirical literature on lung transplantation. Future research should prioritize longitudinal designs capable of capturing psychological trajectories across pre-transplant, acute postoperative, and long-term phases. Cross-sectional studies, while informative, are insufficient to elucidate causal pathways and temporal interactions between medical events and psychological adaptation. The proposed framework should be considered hypothesis-generating and requires prospective empirical validation.

Future validation studies could examine longitudinal changes in psychological distress, health-related quality of life, adherence, self-management, return to work, and healthcare utilization as primary outcomes. Potential mediating mechanisms include ICU-related psychological experiences, identity reconstruction, relational trust, and illness uncertainty, whereas caregiver support, health literacy, medical complications, and continuity of psychosocial care may function as moderators of adaptation trajectories.

There is also a need for more nuanced operationalization of adherence, relational trust, and fear of rejection as dynamic constructs rather than static endpoints. Integrating patient-reported outcomes with clinical indicators may allow for a more ecologically valid understanding of transplant success.

Mixed-methods approaches, combining quantitative assessment with qualitative exploration of patient narratives, are particularly well suited to this field. Conceptual framework development in complex clinical phenomena requires integration of heterogeneous sources of knowledge rather than reliance on a single methodological tradition [9,10]. Such methodologies can illuminate processes of identity reconstruction, meaning-making, and relational adaptation that remain underrepresented in purely quantitative paradigms.

Furthermore, intervention studies evaluating structured psychosocial care pathways—rather than isolated therapeutic techniques—are needed to determine whether integrated models influence long-term outcomes, including quality of life, adherence, and healthcare utilization. Preliminary evidence from collaborative care interventions in transplant populations suggests beneficial effects on symptom burden and healthcare use, supporting the feasibility of testing integrated psychosocial pathways prospectively [73].

Compared with existing biopsychosocial, coping, and quality-of-life frameworks, SPA-LT introduces a stage-based conceptualization of psychosocial adaptation specifically tailored to lung transplantation. The model integrates psychosocial assessment, postoperative neurocognitive vulnerability, long-term identity reconstruction, and continuity of care within a single longitudinal framework. To the author’s knowledge, no previous model has combined these elements in a transplant-specific structure spanning the entire lung transplant trajectory.

### 9.3. Limitations

This article has several limitations. First, it is a conceptual paper based on a narrative synthesis of selected empirical, theoretical, and clinical literature rather than a systematic review. Therefore, it does not aim to provide an exhaustive summary of all available studies on psychosocial outcomes after lung transplantation. Second, some elements of the proposed framework are informed by evidence from broader solid organ transplantation, chronic illness, and intensive care literature, which may not fully capture the specific features of lung transplantation. Third, the proposed stage-based psychosocial adaptation model has not yet been empirically validated. Its clinical usefulness, predictive value, and impact on patient-reported and clinical outcomes require prospective evaluation. As a single-author conceptual framework, the model reflects one clinically informed synthesis and should be further refined through multidisciplinary expert consensus and patient involvement. Finally, implementation of the framework may vary across transplant centers depending on available psychosocial resources, team structure, and local models of care.

## 10. Conclusions

Lung transplantation constitutes not only a complex surgical intervention but also a prolonged psychosocial process embedded within chronic medical uncertainty. Existing conceptual models have provided valuable foundations; however, they often lack temporal sensitivity and insufficiently account for the medically driven discontinuities characteristic of this trajectory.

This article has integrated current empirical and theoretical knowledge to propose a longitudinal psychosocial adaptation framework for lung transplantation. The framework links pre-transplant vulnerability, acute postoperative instability, and long-term adjustment within a coherent structure of psychosocial care. By emphasizing continuity, stage sensitivity, and relational integration, it seeks to move beyond fragmented assessment toward systematic, clinically embedded psychosocial support.

Conceptual synthesis approaches are particularly suited to areas where heterogeneous clinical and psychological evidence must be organized into clinically usable structures [9,10]. Within this perspective, the proposed framework should be understood not as a replacement for empirical research but as a theoretical scaffold guiding future hypothesis testing and intervention development.

Crucially, psychological outcomes should be reframed as core transplant endpoints rather than peripheral variables. Emotional functioning, relational trust, and patient-reported quality of life are not secondary to graft survival; they shape the lived experience of transplantation and influence long-term engagement with care.

Systematic integration of psychosocial expertise within transplant teams may represent an essential component of comprehensive transplant medicine. Recognizing patients as active partners navigating a medically and existentially complex journey allows transplant care to align surgical success with psychological sustainability.

By introducing a stage-based psychosocial adaptation model, this article shifts the focus from static risk stratification toward a longitudinal understanding of psychological processes as integral components of transplant outcomes.

The principal contribution of SPA-LT is not the introduction of new psychosocial constructs but the integration of existing evidence into a longitudinal, clinically oriented framework specifically tailored to lung transplantation. Unlike previous conceptual approaches, SPA-LT explicitly links stage-specific psychosocial assessment, intervention planning, postoperative neurocognitive vulnerability, identity reconstruction, and continuity of care across the transplant trajectory. Future prospective studies should evaluate whether stage-specific psychosocial pathways derived from this framework are associated with improved quality of life, adherence, relational trust, healthcare utilization, and clinical outcomes after lung transplantation.

## Figures and Tables

**Table 1:** Pre-transplant psychosocial domains in lung transplantation: clinical meaning and intervention targets.

Psychosocial Domain	Clinical Meaning in Lung Transplant Candidates	Potential Intervention Targets	Possible Associated Clinical Implications
Emotional distress (anxiety, depressive symptoms)	Context-dependent response to dyspnea, uncertainty, and existential threat; may fluctuate with medical status	Psychoeducation, anxiety regulation strategies, brief CBT-based interventions, meaning-focused support	Persistent anxiety, reduced engagement, potential nonadherence
Decisional ambivalence	Coexistence of hope and fear regarding transplantation; moral and existential tension	Motivational interviewing, values clarification, structured decision support	Delayed decision-making, reduced treatment engagement
Identity disruption	Loss of former roles and autonomy; perceived shift from “person” to “patient”	Narrative-based interventions, identity reconstruction work, role redefinition support	Withdrawal, reduced motivation, difficulty engaging in rehabilitation
Anticipatory loss	Grief for functional capacity, independence, and imagined future	Grief-informed counseling, normalization of anticipatory emotions	Depressive symptoms, hopelessness, disengagement
Health behaviors (nicotine, alcohol use)	Coping attempts, emotion regulation deficits, or embedded lifestyle patterns	Behavioral change support, addiction counseling, stage-matched motivational work	Continued substance use, increased perioperative risk
Adherence-related vulnerability	Cognitive overload, depressive inertia, limited health literacy, chaotic life context	Simplified regimens, adherence coaching, caregiver involvement, structured follow-up	Nonadherence, increased risk of graft complications
Social support and environmental stability	Protective or risk factor influencing emotional regulation and practical functioning	Family-based psychoeducation, resource mapping, social work integration	Poor self-management, increased caregiver burden, worse outcomes
Psychological resilience and coping style	Individual differences in adaptation capacity	Strength-based interventions, resilience training, stress inoculation approaches	Maladaptive coping, emotional dysregulation

**Table 2:** Early post-transplant psychosocial domains.

Psychosocial Domain	Clinical Meaning	Intervention Targets	Possible Associated Clinical Implications
Delirium and confusion	Neurocognitive instability due to ICU factors, sedation, hypoxia	Delirium prevention protocols, orientation strategies, staff communication	Prolonged ICU stay, long-term cognitive impairment
Acute stress reactions	Context-dependent fear, agitation, dissociation	Emotional containment, validation, brief supportive interventions	Persistent distress, risk of PTSD-like symptoms
ICU-related distress	Fragmented memory, sensory overload, loss of temporal continuity	ICU diaries, early psychological presence, post-ICU debriefing	Post-intensive care syndrome, trauma-related symptoms
Loss of agency	Dependence, inability to communicate, restricted autonomy	Restoring autonomy, involving patient in small decisions	Learned helplessness, reduced engagement in recovery
Bodily alienation	Perception of transplanted organ as foreign	Normalization, narrative integration, psychoeducation	Identity disturbance, emotional distress
Early PTSD-like symptoms	Intrusions, hyperarousal, avoidance	Monitoring, early intervention if persistent	Chronic PTSD, impaired quality of life

**Table 3:** Long-term psychosocial domains.

Psychosocial Domain	Clinical Meaning	Intervention Targets	Possible Associated Clinical Implications
Fear of rejection	Chronic vigilance and uncertainty	Psychoeducation, cognitive restructuring, acceptance-based approaches	Persistent anxiety, hypervigilance, reduced quality of life
Emotional distress	Anxiety/depression linked to chronic illness burden	Accessible psychological support, stepped care	Chronic depression/anxiety, reduced adherence, poorer outcomes
Identity reconstruction	Integration of “new self” after transplant	Narrative work, identity-focused interventions	Identity disturbance, reduced life satisfaction, disengagement
Social and relational functioning	Shifts in family roles and relationships	Family interventions, communication support	Relationship strain, caregiver burden, social withdrawal
Return to activity/work	Functional recovery with limitations	Vocational support, gradual reintegration	Reduced participation, loss of role functioning, decreased QoL
Adherence as relational construct	Behavior influenced by trust and alliance	Strengthening therapeutic relationship, shared decision-making	Nonadherence, increased risk of graft complications

**Table 4:** Comparison of Existing Conceptual Frameworks and the Proposed SPA-LT Model.

Framework	Main Focus	Strengths	Limitations in Lung Transplantation	Contribution of SPA-LT
**Biopsychosocial model**	Integration of biological, psychological and social determinants of health	Holistic perspective; recognizes psychosocial influences on health and illness	Lacks temporal organization; provides limited guidance for stage-specific psychosocial care across the transplant trajectory	Introduces temporal organization and stage-specific psychosocial tasks embedded within medical care
**Stress and coping models**	Cognitive appraisal, coping strategies and adaptation to illness	Explains individual differences in psychological adjustment; emphasizes coping resources and resilience	Less applicable during periods of reduced agency (e.g., ICU stay); underestimates medically driven discontinuities	Integrates coping processes with ICU-related vulnerability, changing medical context and longitudinal adaptation
**Quality-of-life frameworks**	Assessment of patient-reported outcomes and functioning	Captures physical, emotional and social outcomes after transplantation	Primarily descriptive; explains outcomes rather than mechanisms of adaptation; limited temporal perspective	Positions HRQoL as one outcome within a broader longitudinal adaptation process
**Psychosocial assessment frameworks (e.g., SIPAT)**	Identification of psychosocial risk before transplantation	Standardized assessment; facilitates multidisciplinary communication; predicts selected outcomes	Frequently used as a gatekeeping tool; limited emphasis on longitudinal intervention planning	Reframes psychosocial assessment as the starting point for individualized, stage-sensitive psychosocial care
**SPA-LT (proposed framework)**	Longitudinal psychosocial adaptation across the entire lung transplant trajectory	Integrates assessment, intervention, continuity of care, postoperative neurocognitive vulnerability, identity reconstruction and long-term adaptation within a transplant-specific clinical pathway	Requires prospective empirical validation and adaptation to local clinical resources	Provides a clinically oriented longitudinal framework intended to guide integrated psychosocial care and generate future research hypotheses

**Table 5:** Operationalization of the Proposed Stage-Based Psychosocial Adaptation Model for Lung Transplantation (SPA-LT).

SPA-LT Stage	Recommended Psychosocial Assessment	Examples of Assessment Tools	Suggested Timing	Potential Intervention Targets	Suggested Outcomes to Monitor
**Pre-transplant**	Psychosocial risk profile; anxiety and depression; coping; health literacy; adherence vulnerability; substance use; social support; resilience	SIPAT, HADS, PHQ-9, GAD-7, Brief COPE, social support questionnaires, clinical interview	Initial transplant evaluation; repeated if clinical status changes or prolonged waiting occurs	Psychoeducation; CBT-based interventions; motivational interviewing; smoking/alcohol cessation; caregiver education; adherence coaching; social work support	Psychological distress; treatment engagement; transplant readiness; adherence; psychosocial stability
**Early postoperative**	Delirium; cognitive functioning; acute stress; ICU-related distress; communication difficulties; emotional adjustment	CAM-ICU, ICDSC, clinical psychological assessment, acute stress screening, cognitive screening where appropriate	ICU stay; step-down unit; before hospital discharge	Delirium prevention; orientation strategies; ICU diaries; supportive psychological care; normalization of emotional responses; family involvement	Delirium resolution; cognitive recovery; PTSD symptoms; rehabilitation engagement; emotional stabilization
**Long-term follow-up**	Quality of life; fear of rejection; adherence; identity reconstruction; social functioning; work participation; caregiver burden; psychiatric symptoms	HRQoL questionnaires (e.g., SF-36, EQ-5D), transplant-specific QoL measures where available, adherence assessment, HADS/PHQ-9/GAD-7, clinical interview	Routine outpatient follow-up; after major complications; annually or when clinically indicated	Stepped psychosocial care; psychotherapy when indicated; vocational support; family interventions; self-management support; psychiatric referral when necessary	HRQoL; adherence; emotional functioning; return to work; self-management; social participation; long-term psychosocial adaptation

## Data Availability

No new data were created or analyzed in this study.

## Abbreviations

LTx	lung transplantation
QoL	quality of life
HRQoL	health-related quality of life
ICU	intensive care unit
PTSD	post-traumatic stress disorder
SIPAT	Stanford Integrated Psychosocial Assessment for Transplantation
CBT	cognitive behavioral therapy
